# Biomarkers of Exposure and Potential Harm in Exclusive Users of Nicotine Pouches and Current, Former, and Never Smokers: Protocol for a Cross-sectional Clinical Study

**DOI:** 10.2196/39785

**Published:** 2022-10-06

**Authors:** David Azzopardi, Linsey Ellen Haswell, Justin Frosina, Michael McEwan, Nathan Gale, Jesse Thissen, Filimon Meichanetzidis, George Hardie

**Affiliations:** 1 British American Tobacco (Investments) Limited Southampton United Kingdom

**Keywords:** biomarkers of exposure, biomarkers of potential harm, nicotine pouches, tobacco harm reduction, cross-sectional clinical study

## Abstract

**Background:**

Tobacco harm reduction (THR) aims to reduce the health burden of cigarettes by encouraging smokers to switch to using alternative tobacco or nicotine products. Nicotine pouches (NPs) are smokeless, tobacco-free, oral products that may be beneficial as part of a THR strategy.

**Objective:**

This 2-center, cross-sectional confinement study conducted in Denmark and Sweden aimed to determine whether biomarkers of exposure (BoEs) to tobacco toxicants and biomarkers of potential harm (BoPHs) in exclusive users of NPs show favorable differences compared with current smokers.

**Methods:**

Participants were healthy NP users (target n=100) and current, former, or never smokers (target n=40 each), as confirmed by urinary cotinine and exhaled carbon monoxide concentrations. During a 24-hour confinement period, participants were asked to use their usual product (NP or cigarette) as normal, and BoEs and BoPHs were measured in blood and 24-hour urine samples, with compliance determined using anabasine, anatabine, and N-(2-cyanoethyl)valine. BoEs and BoPHs were compared between NP users and current, former, and never smokers. Urinary total 4-(methylnitrosamino)-1-(3-pyridyl)-1-butanol (BoE to nicotine-derived nitrosamine ketone) and urinary 8-epi-prostaglandin F2α type III, exhaled nitric oxide, blood carboxyhemoglobin, white blood cell count, soluble intercellular adhesion molecule-1, and high-density lipoprotein cholesterol (BoPHs) were evaluated as primary outcomes. Other measures included urinary 11-dehydrothromboxane B2, forced expiratory volume, carotid intima-media thickness, self-reported quality of life, and oral health.

**Results:**

The results of this study were received in mid-2022 and will be published in late 2022 to early 2023.

**Conclusions:**

The results of this study will provide information on toxicant exposure and biomarkers associated with the development of smoking-related diseases among users of NPs compared with smokers, as well as on the potential role of NPs in THR.

**Trial Registration:**

International Standard Randomised Controlled Trial Number (ISRCTN) ISRCTN16988167; https://www.isrctn.com/ISRCTN16988167

**International Registered Report Identifier (IRRID):**

DERR1-10.2196/39785

## Introduction

### Background

Cigarette smoking is associated with several health risks, including the development of lung cancer and cardiovascular disease [1]. Although the addictive properties of cigarette smoking are primarily due to the tobacco constituent nicotine [2,3], its disease mechanisms, including DNA damage and oxidative stress [4,5], are associated with the long-term inhalation of smoke from the combusted tobacco [1,6]. This knowledge has led to the concept of tobacco harm reduction (THR), whereby smokers are encouraged to replace cigarette smoking with the use of alternative nicotine products with potentially fewer health risks [7]. Such an approach might reduce the health burden of tobacco use [8] and is currently supported by a number of health and regulatory bodies [9-11], although THR is not universally implemented or accepted [12]. Furthermore, for THR to realize its full potential, complete switching from the more harmful product, typically tobacco cigarettes, to the less harmless product is required [13].

**Figure 1:**
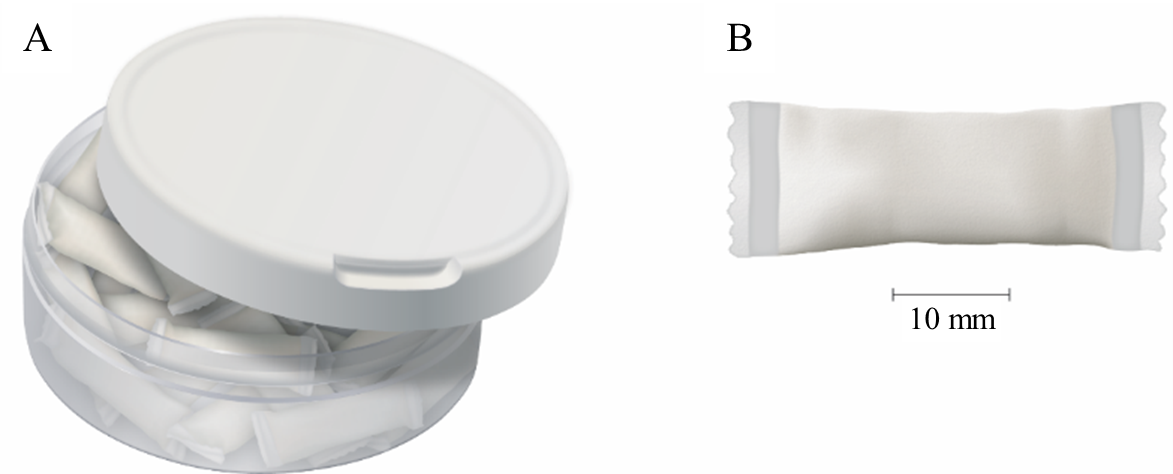
Illustration of a typical nicotine pouch: (A) as sold in container with lid, and (B) individual pouch. It is from “Chemical characterization of tobacco-free “modern” oral nicotine pouches and their position on the toxicant and risk continuums” by David Azzopardi, Chuan Liu & James Murphy David Azzopardi, Chuan Liu & James Murphy (2021) taken from Drug and Chemical Toxicology (2022), Vol45:5, Informa UK Limited, trading as Taylor & Francis Group (2022), reprinted by permission of the publisher.

Commercially available since the mid-2010s, nicotine pouches (NPs; Figure 1) are nicotine-containing oral, smokeless, tobacco-free pouches [14,15]. Although NPs are growing in popularity [16,17], use of NPs is relatively low; in a representative monthly survey of British adults conducted between November 2020 and October 2021, only 0.26% used NPs [18]. Similar to Swedish snus, which is a smokeless tobacco product that has been recognized to have reduced health risks compared with combustible cigarettes [19,20], NPs are placed between the gum and top lip, where nicotine is released from the cellulose matrix in the pouch and absorbed through the oral mucosa.

Because of their relatively simple composition, that is, pharmaceutical-grade nicotine added to a cellulose-based matrix rather than a nicotine-containing tobacco matrix, NPs contain fewer toxicants compared with snus and may therefore present similar or fewer health risks. This has been demonstrated in a recent toxicant analysis in which 24 to 26 compounds (23 to 25 of which were harmful and potentially harmful constituents [HPHCs]) were measured in snus, NPs, and a nicotine replacement therapy (NRT) gum and lozenge. In total, 22 out of 25 of the measured HPHCs were not quantified in the NPs, whereas only 11 out of 23 HPHCs were not quantified in Swedish snus [15]. In addition, studies have demonstrated that extracts from NPs are significantly less toxicologically active in vitro than extracts from Swedish snus or cigarette smoke [21,22].

The aforementioned findings indicate that NPs may have a potential role to play in a THR approach, as recently suggested by Palmer et al [23]. However, at present, there are no data on a user’s actual exposure to toxicants from these products. It should be noted that a reduction in toxicant exposure from alternative nicotine product use compared with continued smoking may not correspond to a reduction in overall harm, and further studies on longer-term use of these products are required to support this. In this regard, clinical studies measuring biomarkers in human samples can provide information on whether NP users are exposed to reduced levels of toxicants and whether this translates to a potential for reduced risk compared with continued smoking. In particular, biomarkers of exposure (BoEs), which indicate a user’s internal exposure to tobacco toxicants, and biomarkers of potential harm (BoPHs), which reflect changes in their wider biological system [24], are now being used to evaluate the risk reduction potential of e-cigarettes and tobacco heating products [25-29].

### Objectives

The aim of this study was to assess whether the lower number and levels of toxicants found in NPs compared with those found in tobacco smoke translate to lower levels of selected BoEs as well as favorable differences in selected BoPHs and physiological measures of health between adults who use NPs and adult current, former, and never smokers.

The primary objective was to quantitatively assess differences between NP users and current smokers in one BoE (total 4-[methylnitrosamino]-1-[3-pyridyl]-1-butanol [NNAL]) and six BoPHs (fractional exhaled nitric oxide [FeNO], 8-epi-prostaglandin F2α type III [8-epi-PGF2α type III], carboxyhemoglobin [COHb], white blood cell (WBC) count, soluble intercellular adhesion molecule-1 [sICAM-1], and high-density lipoprotein [HDL]).

The secondary objectives were to quantitatively assess differences between NP users and current smokers in the BoEs’ total nicotine equivalents (nicotine, cotinine, 3-hydroxycotinine, and their glucuronide conjugates); monohydroxybutenylmercapturic acid; 3-hydroxy-1-methylpropylmercapuric acid (HMPMA); 3-hydroxypropylmercapturic acid; total N-nitrosonornicotine; 3-hydroxybenzo[a]pyrene; S-phenylmercapturic acid; the BoPH 11-dehydrothromboxane B2 (11-dTX B2); the physiological measures forced expiratory volume in 1 second as percentage of predicted (FEV_1_%pred), carotid intima-media thickness (CIMT), and oral health; and a quality-of-life questionnaire. In addition, all study end points were compared between NP users and former smokers and between NP users or former smokers and never smokers.

## Methods

### Study Design

This cross-sectional multicenter confinement study was conducted among exclusive NP users and current, former, and never smokers attending 1 of 2 centers in Herlev, Denmark, and Uppsala, Sweden, between March 2021 and January 2022. Written informed consent was obtained from all participants before screening and enrollment.

### Ethics Approval

Ethics approval for the study was obtained from the Scientific Ethics Committees for the Capital Region, Denmark (H-21021424) and the Ethical Review Authority, Sweden (2021-01810). The study was conducted in accordance with the Declaration of Helsinki and will be reported based on the guidelines of the International Council on Harmonisation. The trial has been registered on the ISRCTN registry (ISRCTN16988167).

### Biomarker Selection

The BoEs chosen for analysis are based on the 9 priority smoke toxicants recommended for mandatory lowering by the World Health Organization [30], which provide an indication of exposure to toxicants present in the gas and particulate phases of tobacco smoke (Table 1). For two of these toxicants (acetaldehyde and formaldehyde), there are no reliable BoEs at present; therefore, levels of crotonaldehyde were assessed (through HMPMA) instead. The BoPHs 11-dTX B2 [31-33], 8-epi-PGF2α type III [34,35], CIMT [36-38], COHb [39-41], FeNO [35,42], FEV_1_%pred [43], HDL [44-46], sICAM-1 [47-49], and WBC count [50-53] were selected to cover a range of associated smoking-related diseases, including lung cancer, cardiovascular disease, and chronic obstructive pulmonary disease, as well as underlying disease processes such as oxidative stress (Table 2). Note that urinary NNAL, a biomarker for nicotine-derived nitrosamine ketone exposure, is also considered a BoPH associated with lung cancer [54,55].

**Table 1:** Biomarkers of exposure measured in the study.

Biomarker of exposure				Associated toxicant			Matrix		Method		References
**Primary end point**											
	Total NNAL^a^				NNK^b^			24-hour urine		LC-MS/MS^c^	[54-57]
**Secondary end points**											
		3-HPMA^d^	Acrolein			24-hour urine			LC-MS/MS		[56,58]
		3-OH-B[a]P^e^	B[a]P^f^			24-hour urine			LC-MS/MS		[59]
		HMPMA^g^	Crotonaldehyde			24-hour urine			LC-MS/MS		[56,58]
		MHBMA^h^	1,3-Butadiene			24-hour urine			LC-MS/MS		[56,58]
		S-PMA^i^	Benzene			24-hour urine			LC-MS/MS		[58]
		TNeq^j^	Nicotine			24-hour urine			LC-MS/MS		[56]
		Total NNN^k^	NNN			24-hour urine			LC-MS/MS		[56]

^a^NNAL: 4-(methylnitrosamino)-1-(3-pyridyl)-1-butanol. Urinary 4-(methylnitrosamino)-1-(3-pyridyl)-1-butanol is associated with lung cancer risk [57]; therefore, it is also considered a biomarker of potential harm for lung cancer [54,55].

^b^NNK: nicotine-derived nitrosamine ketone.

^c^LC-MS/MS: liquid chromatography with tandem mass spectrometry.

^d^3-HPMA: 3-hydroxypropylmercapturic acid.

^e^3-OH-B[a]P: 3-hydroxybenzo[a]pyrene.

^f^B[a]P: benzo[a]pyrene.

^g^HMPMA: 3-hydroxy-1-methylpropylmercapuric acid.

^h^MHBMA: monohydroxybutenylmercapturic acid.

^i^S-PMA: S-phenylmercapturic acid.

^j^TNeq: total nicotine equivalents (nicotine, cotinine, 3-hydroxycotinine, and their glucuronide conjugates).

^k^NNN: N-nitrosonornicotine.

**Table 2:** Biomarkers of potential harm measured in the study.

Biomarker of potential harm				Associated biological process		Matrix		Method	References
**Primary end points**									
	8-Epi-PGF2α type III^a^		Oxidative stress		24-hour urine		LC-MS/MS^b^		[34,35,60]
	COHb^c^		CVD^d^		Whole blood		HS GC-MS^e^		[39-41,61]
	FeNO^f^		Airway inflammation		Exhaled breath		Chemical field-effect transistor		[35,42,62]
	HDL^g^		CVD		Blood		Enzyme colorimetric		[28,44-46]
	sICAM-1^h^		CVD		Serum		ELISA^i^		[28,47-49]
	WBC^j^ count		Inflammation		Blood		Flow cytometry		[50-53,63]
**Secondary end points**									
		11-dTX B2^k^		CVD		24-hour urine		LC-MS/MS	[31-33,60]
		CIMT^l^		CVD		Physiological measurement		Ultrasound	[36-38,64]
		FEV_1_%pred^m^		COPD^n^		Physiological measurement		Spirometry	[43,65,66]

^a^8-Epi-PGF2α type III: 8-Epi-prostaglandin F2α type III.

^b^LC-MS/MS: liquid chromatography with tandem mass spectrometry.

^c^COHb: carboxyhemoglobin.

^d^CVD: cardiovascular disease.

^e^HS GC-MS: headspace gas chromatography–mass spectrometry.

^f^FeNO: fractional exhaled nitric oxide.

^g^HDL: high-density lipoprotein.

^h^sICAM-1: soluble intercellular adhesion molecule-1.

^i^ELISA: enzyme-linked immunosorbent assay.

^j^WBC: white blood cell.

^k^11-dTX B2: 11-dehydrothromboxane B2.

^l^CIMT: carotid intima-media thickness.

^m^FEV_1_%pred: forced expiratory volume in 1 second as percentage of predicted.

^n^COPD: chronic obstructive pulmonary disease.

### Study Participants

All participants were healthy adult men or women aged 19 to 55 years. For all other inclusion and exclusion criteria, refer to Textbox 1. Exclusive NP users as well as current, former, and never smokers were recruited in Sweden and Denmark, with an equal split of Swedish and Danish participants in each arm. Participants were selected either from a database of individuals who were registered at the participating clinics for the purpose of undertaking clinical studies or through study-specific advertising (eg, social media). Because of difficulties in recruiting NP users by the study clinics alone, an external recruitment agency assisted in identifying potential participants from its database and referred interested individuals to the study clinics without releasing any personal information.

For the NP user group, participants were self-reported solus users of at least three Lyft NPs (currently marketed as Velo; British American Tobacco) per day and had used these NPs for a minimum of 6 months before screening. For the current smoker group, participants were self-reported solus smokers of at least 10 factory-made cigarettes (FMCs) per day and had smoked for at least one year before screening. For the former smoker group, participants were self-reported former smokers of FMCs who quit smoking at least six months before screening. For the never smoker group, participants had never smoked (<100 cigarettes in their life and none within the 6 months before screening). Compliance with long-term smoking abstinence in the NP and former smoker groups was verified by analysis of N-(2-cyanoethyl)valine (CEVal) in erythrocytes [67]. Urinary levels of anabasine (AB) and anabatine (AT) were also used to determine short-term abstinence from smokeless tobacco use.

**Inclusion and exclusion criteria.**

**Inclusion criteria**

- Participants who are healthy men or women, aged 19 to 55 years
- Participants who have a BMI of 18.5 to 30.0 kg/m^2^ (body weight exceeding 52 kg [men] or 45 kg [women])
- Participants who are in good health as judged by the principal investigator (PI) or the appropriately qualified designee based on medical history, physical examination, vital signs assessment, 12-lead electrocardiogram, clinical laboratory evaluations, and lung function spirometry test
- Participants who have given their written informed consent to participate in the study and have agreed to abide by the study restrictions
- Participants who can demonstrate the ability to comprehend the informed consent form, are able to communicate well with the PI or the appropriately qualified designee, can understand and comply with the requirements of the study, and can be judged suitable for the study in the opinion of the PI or the appropriately qualified designee
- Participants who will refrain from consuming alcohol within 24 hours before screening and admission
- Participants who will refrain from consuming cruciferous vegetables as well as grilled, fried, or barbequed food and avoid being in the presence of the cooking of cruciferous vegetables as well as grilled, fried, or barbequed food for 48 hours before screening and admission
- Arm A: exclusive nicotine pouch (NP) users
  Participants who are regular (daily) users of at least three Lyft NPs (British American Tobacco) per day
  Participants who have used Lyft for a minimum of 6 months before screening
  Participants who have a urinary cotinine level ≥200 ng/mL and an exhaled carbon monoxide (eCO) level <7 ppm at screening and admission
- Arm B: current smokers
  Participants who are regular solus smokers of commercially manufactured filter cigarettes
  Participants who have smoked for at least one year before screening
  Participants who typically smoke at least 10 cigarettes per day and have a urinary cotinine level ≥200ng/mL and an eCO level ≥7 ppm at screening and admission
- Arm C: former smokers
  Participants who are former smokers of commercially manufactured filter cigarettes who quit smoking at least six months before screening
  Participants who have a urinary cotinine level <200 ng/mL and an eCO level <7 ppm at screening and admission
- Arm D: never smokers
  Participants who have never smoked (<100 cigarettes in their life and none within the 6 months before screening)
  Participants who have a urinary cotinine level <200 ng/mL and an eCO level <7 ppm at screening and admission

**Exclusion criteria**

- Female participants who are pregnant or breastfeeding (confirmed at screening)
- Participants who have donated ≥400 mL of blood within 90 days before screening, plasma in the 7 days before screening, and platelets in the 6 weeks before screening
- Participants who have had an acute illness (eg, upper respiratory tract or viral infection) within 4 weeks before screening as judged by the PI
- Participants who have a significant history of alcoholism or drug or chemical abuse (apart from known smoking and vaping history) within 24 months before screening as determined by the PI or the appropriately qualified designee
- Participants who have a positive urine drugs of abuse panel or breath alcohol screen result (confirmed by repeat) at screening or admission
- Participants who have serum hepatitis or are carriers of the hepatitis B surface antigen or are carriers of the hepatitis C antibody; have a positive result for the test for HIV antibodies; have symptoms of a COVID-19 infection, have a positive result in the COVID-19 test at screening or admission indicating current, active infection (Sweden only), or not providing proof of a negative COVID-19 test taken within 48 hours of admission (Denmark only)
- Participants who have used prescription or over-the-counter bronchodilator medication (eg, inhaled or oral β-adrenergic agonists) to treat a chronic condition within the 12 months before screening
- Participants who have received any medications or substances (other than nicotine) that interfere with the cyclooxygenase pathway (eg, anti-inflammatory drugs, including aspirin and ibuprofen) within 14 days before screening or are known to be strong inducers or inhibitors of cytochrome P450 enzymes within 14 days or 5 half-lives of the drug (whichever is longer) before screening
- Participants who would need to take prescription medication not approved by the PI during the period beginning with screening and ending with discharge (for female participants, hormonal contraceptives are acceptable, and for all participants, painkillers [eg, paracetamol] are permitted)
- Participants who are unwilling or unable to comply with the study requirements
- Employees and immediate relatives of the tobacco industry or the clinical site
- Participants who have any clinically relevant abnormal findings on the physical examination, medical history, electrocardiogram, lung function tests, or clinical laboratory panel, unless deemed not clinically significant by the PI or the appropriately qualified designee
- Participants who have been diagnosed with a significant history of urticaria or asthma (childhood asthma is acceptable)
- Participants who have, or who have a history of, any clinically significant neurological, gastrointestinal, renal (including urinary tract infection or nephrolithiasis), hepatic, cardiovascular, psychiatric, respiratory, metabolic, endocrine, hematological, or other major disorder that, in the opinion of the PI or the appropriately qualified designee, would jeopardize the safety of the participant or have an impact on the validity of the study results
- Participants who have previously been diagnosed with any form of malignancy or carcinoma in situ
- Participants who are currently participating in another clinical trial (including follow-up)
- Participants who, in the opinion of the PI or the appropriately qualified designee, should not participate in this study
- Arm A: exclusive NP users
  Participants who have used any form of tobacco or nicotine-containing product, other than the Lyft NP products, within 6 months before screening
- Arm B: current smokers
  Participants who are self-reported noninhalers (smokers who draw smoke from the cigarette into the mouth and throat but who do not inhale)
- Arms C and D: former and never smokers
  Participants who have used any form of tobacco or nicotine-containing product within the 6 months before screening

### Investigational Products

No investigational products were provided; instead, participants were required to bring their own supply of NPs or cigarettes for use during the study confinement period. However, all participants recruited to the NP group were self-reported solus users of Lyft NPs; those recruited to the current smoker group could be smokers of any brand of FMCs.

### Study Procedures

Potential participants were invited to attend the clinic to assess study eligibility. They received verbal and written information about the study and were asked to sign the informed consent form before undergoing any procedures. Screening consisted of physical, oral, and vital signs examinations, as well as routine laboratory testing. Tests for alcohol and drug consumption as well as pregnancy (women only) were also conducted, and extent of nicotine use and smoking status were determined through a questionnaire.

Individuals deemed eligible based on the screening assessments were invited back to the clinic for admission into the study within 7 days of screening. They were asked to bring with them a sufficient supply of their usual NPs (Lyft brand) or usual cigarettes to last the whole screening and confinement period. In addition, NP users were asked to bring excess pouches to enable the analysis of unused pouches for nicotine content. Those who successfully completed the admission assessments (Table 3) were enrolled in the study (day 1) and given a unique study number. Participant data were collected on a paper case report form (CRF) or entered directly on an electronic case report form (eCRF). After enrollment, participants began a period of 24-hour urine collection and were confined to the clinic during this time. The remaining study assessments, including blood sampling and physiological assessments, were performed during the remainder of the confinement period before discharge on day 2 (Table 3). During the confinement period, participants were requested to use their NPs or cigarettes as they would normally, except where product use might interfere with on-study assessments. This approach facilitated the measurement of short-term BoEs that reflected the participant’s product use outside the clinic. In the case of NP users, pouches were collected after use for assessment of residual nicotine content. On 3 random occasions, participants in the NP group were asked to record the duration of pouch use to the nearest minute. They commenced timing upon placing the pouch under the lip and ended timing on removal of the pouch. Participants were discharged from the clinic on day 2 after completion of both the 24-hour urine collection period and study and discharge assessments. No later than 1 week after discharge, a poststudy follow-up was performed by telephone call to collect information on the status of any ongoing adverse events (AEs) at discharge and any new AEs experienced after discharge.

**Table 3:** Schedule of assessments.

Assessment	Screening	Admission (day 1)	On study (days 1-2)	Discharge (day 2)	Poststudy follow-up^a^
Participants are free to use NP^b^ or smoke as usual^c^	✓	✓	✓	✓	✓
Participants collect used NPs^d^			✓		
Unused pouch collection			✓		
Informed consent	✓				
Inclusion and exclusion criteria	✓				
Sociodemographic data	✓				
Medical history	✓				
Prior and concomitant medications	✓	✓	✓	✓	✓
Tobacco and nicotine use history questionnaire	✓				
Pregnancy test (urine and serum)^e^	✓	✓			
COVID-19 test (Sweden only)	✓	✓			
Height, weight, BMI, and waist circumference	✓				
Vital signs^f^	✓	✓			
12-lead ECG^g^	✓				
Physical examination^h^	✓	✓		✓	
Urinary cotinine screen (dipstick)	✓	✓			
Urine drugs of abuse panel and alcohol screen^i^	✓	✓			
Serum biochemistry and hematology	✓				
Urinalysis	✓				
Virology (hepatitis B and C and HIV)	✓				
Exhaled carbon monoxide measurement^j^	✓	✓			
Spirometry (without bronchodilator)^k^	✓				
FeNO^l^ measurement^m^			✓		
24-hour urine collection			✓		
Blood sampling for biomarker analysis^n^			✓		
Carotid intima-media thickness assessment			✓		
Quality-of-life questionnaire			✓		
Oral health assessment			✓		
Adverse event recording^o^	✓	✓	✓	✓	✓

^a^Within 7 days of discharge.

^b^NP: nicotine pouch.

^c^At any time, unless it would interfere with study assessments.

^d^All pouches used during confinement collected from participants in arm A commencing just before the start of 24-hour urine collection.

^e^Urine pregnancy test only at admission.

^f^Includes pulse rate, systolic and diastolic blood pressure, respiratory rate, and tympanic temperature.

^g^ECG: electrocardiogram.

^h^Full physical examination at screening; symptom-driven physical examination at admission and discharge, if deemed necessary.

^i^By breath test (alcometer).

^j^No food, smoking, or nicotine pouch use within 30 minutes before assessment.

^k^No food within 2 hours before assessment; no smoking or nicotine pouch use within 1 hour before assessment.

^l^FeNO: fractional exhaled nitric oxide.

^m^No food or drink within 1 hour before assessment; no smoking or nicotine pouch use within 30 minutes before assessment.

^n^Blood sampling for the following biomarker analysis: N-(2-cyanoethyl)valine, carboxyhemoglobin (drawn between 6 PM and 8 PM), lipid panel (for high-density lipoprotein analysis), soluble intercellular adhesion molecule-1, and white blood cell count.

^o^Reporting begins at provision of informed consent.

At any point, participants were able to withdraw from the study for any reason, or they may have been withdrawn at the discretion of the principal investigator (PI) or study sponsor (eg, for health reasons or protocol deviations). The reason for premature discontinuation would be clearly documented in the participant’s eCRF. The PI could suspend or terminate the study for any reason after consultation with the sponsor; the sponsor could also suspend or terminate the study for any reason. If the study was terminated, the reasons would be fully documented.

### Study Assessments

#### Overview

Routine clinical laboratory testing was conducted at screening to exclude individuals with significant medical conditions. Urine collection began on day 1, immediately after enrollment; all urine voided was pooled at the end of the 24-hour period and mixed before analyses. Blood samples were obtained on days 1 and 2 through direct venipuncture or a cannula inserted in a forearm vein. The blood sample for COHb analysis was drawn between 6 PM and 8 PM. A maximum of 100 mL was drawn and used for both laboratory tests and biomarker evaluations. Physiological assessments were performed on days 1 and 2 before discharge.

#### Compliance

For participants in the NP and former smoker groups, compliance with long-term abstinence from smoking before the study was assessed by measurement of CEVal in erythrocytes derived from 5 mL of whole blood [68]. In addition, short-term abstinence from smokeless tobacco use was assessed by measurement of urinary AB and AT. CEVal, AB, and AT were measured at Analytisch-Biologisches Forschungslabor (ABF) in Munich, Germany. AB and AT were analyzed by LC-MS/MS as follows. In brief, 60 µL deuterated internal standard (IS) solutions (AB-D4 and AT-D4, 12 ng each) and 900 µL formic acid were added to 0.6 mL urine and the mixture subjected to solid phase extraction on Oasis MCX cartridges (60 mg, 3 mL; Waters). After washing with 1.8 mL 0.5% formic acid, 3.2 mL water, 1.8 mL methanol, and 1.8 mL acetonitrile/methanol (6:4, volume:volume), and elution with 1.2 mL 2% aqueous ammonium hydroxide/59% acetonitrile/39% methanol, evaporation of the eluate, and reconstitution with 100 µL 10 mM aqueous ammonium acetate/acetonitrile (9:1, volume:volume), 10 µL was injected into an LC-MS/MS system (HP 1100 HPLC [Agilent Technologies] coupled to an API 4000 [Sciex]). Chromatography was conducted with a Gemini C18 (2) column (150×3 mm, 3 µm particle size; Phenomenex) by applying a gradient consisting of 10 mM aqueous ammonium acetate (A) and acetonitrile (B) under the following conditions: 0.5 mL/minute, 50 °C; 0 to 3 minutes: 10% to 75% B; 3 to 4 minutes: 75% B; 4 to 4.1 minutes: 75% to 10% B; and 4.1 to 9 minutes: 10% B. Mass spectrometric analysis was conducted in positive electrospray ionization mode using multiple reaction monitoring. Mass-to-charge ratios (m/z) for quantifier/qualifier transitions: AB: 163→80/163→92, AB-D4: 167→84/–, AT: 161→144/161→107, and AT-D4: 165→148/–. Limit of detection and lower limit of quantification for AB and AT in urine were 0.13/0.39 ng/mL and 0.08/0.24 ng/mL, respectively.

#### Measuring BoEs

Total NNAL [56], total nicotine equivalents [56], monohydroxybutenylmercapturic acid [56,58], HMPMA [56,58], 3-hydroxypropylmercapturic acid [56,58], total N-nitrosonornicotine [56], S-phenylmercapturic acid [58], and 3-hydroxybenzo[a]pyrene [59] in urine were analyzed by ABF using validated LC-MS/MS methods as previously described.

#### Measuring BoPHs

HDL was measured at Nordic Bioscience (Herlev, Denmark) and Clinical Chemistry and Pharmacology laboratory at Uppsala University Hospital in Uppsala, Sweden, using Advia Chemistry System–Direct HDL Cholesterol (Siemens Healthcare) and Cobas Pro (Roche Diagnostics International), in accordance with the manufacturer’s protocol, respectively. Total WBC count was measured at Sanos Clinic (Herlev, Denmark) and Clinical Chemistry and Pharmacology laboratory (Uppsala University Hospital, Uppsala, Sweden) using the XN-550 and XN-20 systems (Sysmex), respectively.

Urinary 8-epi-PGF2α type III and 11-dTX B2 measurements were carried out at ABF as previously described [60]. Plasma sICAM-1 was measured at Celerion (Zurich, Switzerland) by an enzyme-linked immunosorbent assay kit (DuoSet; R&D Systems).

COHb analysis was carried out at ABF by headspace gas chromatography–mass spectrometry as previously described [61], with modifications. In brief, 100 µL of whole blood was spiked with 50 µL of IS solution (saturated whole blood containing 13COHb) and 1.4 mL of water. Carbon monoxide was released with the addition of 200 µL of potassium hexacyanoferrate solution 200 g/L at 55 °C for 30 minutes. Next, 1 mL of the head space was injected into a model 6890 gas chromatograph interfaced to a model 5973 mass selective detector (Agilent Technologies) using a multipurpose autosampler (Gerstel). Chromatographic separation was conducted on an Rt-Msieve 5A porous layer open tubular capillary column (30 m × 0.32 mm inner diameter, 30 µm film thickness; Restek). The injector temperature was set to 150 °C with a split of 9:1 and a constant helium flow of 1.9 mL/minute. An isothermal temperature program (45 °C) was applied for chromatographic separation. Mass spectrometry detection was performed in the selected ion monitoring mode with electron impact ionization. The transfer line temperature was set to 280 °C with a source temperature of 230 °C and a quadrupole temperature of 150 °C. The mass fragment m/z 28 (IS: 29) was used for quantification, with m/z 12 (IS: 13) as qualifier.

For CIMT, ultrasound assessment was performed on a 10-mm section of the distal portion of the common carotid artery, on both sides of the neck, at least 5 mm from the carotid bulb. The mean, SD, and maximum thickness of the intima-media were recorded using the Acuson P500 Ultrasound System (Siemens Healthcare).

FEV_1_%pred was measured by spirometry assessment (without a bronchodilator) using the EasyOne Pro (Sweden) or Easy on-PC (Denmark) spirometers (NDD Medical Technologies) in accordance with the procedures outlined by the American Thoracic Society and European Respiratory Society [65]; values were standardized to predictive values of the Global Lung Function Initiative [66]. Participants were not allowed to eat for 2 hours and 1 hour, or to use NPs or smoke for 1 hour and 30 minutes, before spirometry and FeNO assessments, respectively. FeNO was measured using the Vivatmo Pro device (Bosch Healthcare Solutions).

#### Other Assessments

Oral health was assessed with the Oral Health Assessment Tool [69,70]. Quality of life was assessed with the 36-Item Short Form Health Survey questionnaire (RAND Corporation) [71].

### Nicotine Content in Used NPs

Unused NPs brought by the participants for nicotine content analysis were stored at 2 °C to 8 °C. NPs used during the confinement period were collected into a single container and stored at 2 °C to 8 °C until analysis. The NPs were analyzed for nicotine content by gas chromatography with flame ionization detector at Labstat International Inc in Kitchener, Ontario, Canada. In brief, 3 unused pouches (approximately 2 g) or all of the participants’ used pouches were cut in half, and both the contents and the pouch material were added to an extraction vessel. Next, 5 mL of 2N sodium hydroxide was added to the extraction vessel, which was subsequently sealed and allowed to stand for 15 minutes. Subsequently, 50 mL of an extraction solution of 10 mL of quinoline primary stock (10 g of quinoline accurately weighed into a 250-mL volumetric flask and diluted to volume with methyl t-butyl ether) diluted to volume with methyl t-butyl ether was added to the extraction vessel, which was then sealed. The extraction vessel was shaken in a linear shaker in a horizontal position for 2 hours, after which it was placed in the dark in a vertical position to allow the phases to separate (maximum 2 hours). The organic phase (top layer) was then transferred to an amber autosampler vial.

### Safety

Participant safety during the study was monitored by physical examination, vital signs, 12-lead electrocardiogram, and laboratory assessments, including hematology, virology, biochemistry, and urinalysis. Any AEs or serious AEs were monitored throughout the confinement period and by telephone follow-up up to 1 week after discharge. If the study was stopped because of an AE, it would not be restarted without consultation with the study ethics committee.

All AEs were recorded on the eCRF, coded in accordance with the latest version of the Medical Dictionary for Regulatory Activities, and tabulated by system organ class and preferred term. Severity was classified as mild (does not cause significant discomfort or change in activities of daily living; symptoms are easily tolerated), moderate (causes inconvenience or concern to the participant; interferes with activities of daily living but such activities may be continued), or severe (significantly interferes with activities of daily living to the point where they cannot be continued, or the participant is incapacitated). Numbers and percentages of participants reporting at least one AE, one serious AE, or an AE leading to withdrawal, as well as numbers and percentages of participants with AEs by severity were reported.

Participants who developed an AE at any time during the study, including the period between discharge and follow-up, were followed until assessments had returned to baseline, or the PI had determined that these events were no longer clinically significant. Provided there were no AEs that required further attention, the participant’s involvement in the study was complete. The ethics committee was informed of study completion within 90 days of the last participant’s final study procedures.

### Statistical Analysis

In the absence of any NP biomarker data, the sample size was based on data from former and current smokers for sICAM-1, which shows the most variability in values in the literature. Assuming a ratio of mean values between NP users and smokers of 0.847 and a coefficient of variation of 27.1% to 32.8% based on data from Haswell et al [72], PROC POWER in SAS software (version 9.4; SAS Institute) was used to calculate that 84 to 120 participants would be the minimum needed to demonstrate a statistically significant difference with β=.2 and α=.05. The split between NP users and current smokers was not planned to be equal; therefore, a minimum sample size of 120 was fixed for the 2 groups combined. To allow for attrition and noncompliant NP users, 100 participants was the target to be recruited to the NP user group, 40 to the current smoker group (because the values are less variable and better described in the literature), and 40 to each of the former and never smoker groups to characterize biomarker levels in these groups. If withdrawal from the NP user group led to a substantial drop in sample size, new participants could be recruited to ensure that minimum values were met.

### Data Analysis

The data were analyzed using SAS version 9.4. For continuous variables, the number of participants, mean, SD, median, minimum, and maximum were tabulated by study arm and overall. Categorical variable frequencies (number and percentages) were tabulated by study arm.

The group means of each of the primary end points were compared between participants who were solus users of Lyft NP products (arm A) and participants who were solus conventional cigarette smokers (arm B) in both the per-protocol population and the CEVal-, AB-, and AT-compliant populations. This was performed using a multiple linear regression model with the respective biomarker (Y_j_) as the dependent variable and the arm (X_1_) as the independent variable. The variables age (X_2_), sex (X_3_), and site (X_4_) were added to the model in a stepwise manner and kept in the final model if they showed significance on an α level of .05. A final model for each biomarker (primary end point) was estimated; for example, the final model could differ among the end points because of the stepwise approach. If the assumption of the model was not valid (normally distributed residual data), then the biomarker data were log-transformed, that is, log(Yj). If the data were log-transformed and the residuals remained not normally distributed, the Mann-Whitney *U* test was used to ensure an accurate testing method. To adjust for multiple testing for the primary end points, Bonferroni correction was applied. The α level was divided by 7 (7 primary end points): .05/7=.007143. Because of the adjustments, the 99.286% CIs for the estimated least square means were presented. The same approach was applied for the secondary end points as previously described for the primary end points but without Bonferroni adjustment.

### Data Management

The protocol for data management is described in full in a data management plan, which was finalized before any data were collected. Completeness of the participants’ records, accuracy of recording on the eCRFs, adherence to both the study protocol and good practice guidelines, and progress of enrollment were checked throughout the study by an independent clinical research associate. The eCRFs served as the source documents for reviewing data collection procedures.

Data that were initially collected on paper documents were entered in the electronic data capture system by staff at each clinical site. Data entry underwent quality control checks, and any discrepancies in the database were resolved. After all data validation steps, the PI or designee electronically signed the completed electronic data before database lock. All primary sources and copies of data generated by each study site (eg, data sheets, CRFs, electronic records, correspondence, laboratory records, and photographs) required for construction and evaluation of the study report will be retained in the archives of the 2 study sites for 25 years after study completion.

## Results

The results of this study were received in mid-2022 and will be published in late 2022 to early 2023.

## Discussion

### Overview

NPs, a modern oral nicotine product, have been commercially available in a number of countries since the mid-2010s. Recent surveys of retail sales [17] and product use [16] show that NPs are gaining popularity in the United States, and some authors have suggested that they may form part of a THR approach [23]. To date, however, there are few data available on these relatively new products [14,15,21,22,73-78].

In terms of use and appearance, NPs are very similar to Swedish snus, an oral smokeless tobacco product that has been traditionally used in Scandinavia for more than 100 years. Although overall tobacco product use in Sweden is comparable with that in the rest of Europe, smoking-associated deaths are much lower because most tobacco consumers use snus [19]. This has been termed the “Swedish experience” [79], and the lower risks of harm from snus compared with combustible cigarettes have been recognized by the US Food and Drug Administration [20]. Of note, a recent analysis of the toxicant content of NPs compared with that of snus has demonstrated that most of the measured HPHCs (22 of 25) are unquantifiable in NPs, whereas only approximately half of the measured HPHCs (11 of 23) are unquantifiable in Swedish snus [15], raising the possibility that, similar to Swedish snus, NPs may have reduced health risks compared with cigarette smoking. To determine whether the low number and levels of HPHCs in NPs translate to a reduction in toxicant exposure and potential risk for users compared with cigarette smokers, this cross-sectional study compared BoEs and BoPHs between individuals who have been exclusively using NPs for 6 months and current smokers. The results from this study will provide an indication of the exposure of NP users to tobacco and tobacco smoke toxicants arising from NP use and relative levels compared with current, former, and never smokers. In addition, this study will give an indication of the real-world levels of BoPHs in regular NP users compared with current, former, and never smokers. Furthermore, the study will generally add to the scientific characterization of these relatively new nicotine products with additional subjective, physiological, and behavioral data.

Biomarker studies are frequently used to evaluate exposure to environmental and occupational toxicants and have recently been applied to assess the effects of switching from smoking combustible cigarettes to using alternative nicotine and tobacco products. Reductions in several BoEs and BoPHs have been documented when smokers switch to using e-cigarettes [27,80,81], tobacco heating products [25,26,28,56], and NRT [27], helping to establish the relative health risks of these products. This study will continue to build on data from these other tobacco and nicotine product categories. To the best of our knowledge, this is the first time that BoEs and BoPHs have been measured in NP users, providing information on 17 BoEs and BoPHs in NP users in comparison with current, former, and never smokers. The findings should add to our overall knowledge on NPs by providing the relative levels of exposure to tobacco toxicants as well as an insight into the potential risk from use of these modern tobacco-free NP products compared with cigarette smoking.

### Strengths

This study includes some strengths. First, participants who self-reported as solus NP users and former smokers were confirmed as not having used combustible tobacco products through CEVal assessment [28,67]. Furthermore, as snus use is popular in Scandinavia, particularly in Sweden, an attempt was made to confirm that the NP users and former smokers did not use snus. Although snus is recognized as a reduced risk product [20], it has been shown to contain a higher number of toxicants than NPs [15]. Therefore, snus use could have potentially affected BoEs and BoPHs if participants in the NP group used snus but failed to report this, which was a possibility given the similarities between use and physical characteristics of NPs and snus. Hence, a further compliance assessment of AB and AT was included because CEVal, a biomarker for acrylonitrile exposure, cannot detect snus use. These alkaloids have been suggested as biomarkers to detect tobacco consumption during NRT use [82,83] and were suggested as potential compliance biomarker candidates to detect short-term smokeless tobacco use during this study by experts from a bioanalytical laboratory, although an assessment of these alkaloids in NPs has not been made. A second strength of this study is that it measured BoEs and BoPHs in individuals who are regular users of NPs through their own choice, rather than in individuals who are asked to try the product for a few days [27,81], and should therefore provide information specific to this user group. Third, this cross-sectional approach recruited regular users of NPs, an approach which may better reflect real-world NP use compared with an approach that asks smokers to switch to NPs as part of a longitudinal design. Fourth, this cross-sectional approach limits the chance of participant withdrawals. Fifth, because of the cross-sectional study design, the sample size of 220 is considerably higher than that in some previous product-switching trials [80]. Finally, an additional strength of the study is that the confinement period allowed 24-hour urine samples to be collected (as opposed to spot urine collection) and blood samples to be collected at consistent times to help minimize variability in the biomarker data.

### Limitations

The study also includes some limitations. First, the cross-sectional study design enables the study population to be assessed at a single point in time, but unlike in longitudinal studies, in which the same individuals are assessed multiple times over a longer study period (eg, up to 12 months [26,28]), information about changes over time will not be obtained. On the basis of the results from longitudinal studies, 6 months of NP use was deemed sufficient for changes in BoEs and particularly in BoPHs to have occurred in this study [28]. Second, the cross-sectional design approach means that no baseline assessments were made. Therefore, this may lead to greater variability in the biomarker results because comparisons were made between different populations as opposed to investigating biomarker-level changes within populations as per a longitudinal study approach. Third, some of the BoPHs assessed in this study (eg, WBCs, HDL, and sICAM-1) are not specific biomarkers of smoking-related disorders and may also be influenced by environmental exposure and lifestyle choices such as diet and exercise [84]. Finally, although compliance measures were implemented, these have limitations in terms of their sensitivity to detect tobacco cigarette and smokeless tobacco use, and they cannot detect use of other nicotine products such as e-cigarettes. In addition, participants self-reported product use (quantity and length of time that they have, or have not, been using the products), which cannot be corroborated fully with the current compliance and screening procedures (ie, eCO). Therefore, we cannot guarantee that current and past product use requirements will be fully met. This information is likely to be controlled better in a longitudinal study in which there is a defined switching period and regular contact with the participants.

### Conclusions

With the rising consumer interest in NPs [16,17] and the potential role of these products in a THR approach [23], the results of this study are expected to provide timely information on BoE and BoPH levels among regular and relatively long-term (>6 months) users of NPs. These data will contribute to the growing body of evidence on NPs and their potential as a reduced-risk alternative nicotine source for smokers who fully switch from combustible cigarettes to using these tobacco-free NPs.

## Abbreviations

11-dTX B2: 11-dehydrothromboxane B2
8-Epi-PGF2α type III: 8-Epi-prostaglandin F2α type III
AB: anabasine
ABF: Analytisch-Biologisches Forschungslabor
AE: adverse event
AT: anatabine
BAT: British American Tobacco
BoE: biomarker of exposure
BoPH: biomarker of potential harm
CEVal: N-(2-cyanoethyl)valine
COHb: carboxyhemoglobin
CRF: case report form
eCO: exhaled carbon monoxide
eCRF: electronic case report form
FeNO: fractional exhaled nitric oxide
FEV_1_%pred: forced expiratory volume in 1 second as percentage of predicted
FMC: factory-made cigarette
HDL: high-density lipoprotein
HMPMA: 3-hydroxy-1-methylpropylmercapuric acid
HPHC: harmful and potentially harmful constituent
IS: internal standard
LC-MS/MS: liquid chromatography with tandem mass spectrometry
m/z: mass-to-charge ratio
NNAL: 4-(methylnitrosamino)-1-(3-pyridyl)-1-butanol
NP: nicotine pouch
NRT: nicotine replacement therapy
PI: principal investigator
sICAM-1: soluble intercellular adhesion molecule-1
THR: tobacco harm reduction
WBC: white blood cell

## Data Availability

British American Tobacco is committed to the responsible sharing of data with the wider research community. Data access is administered for this study through an internal data-sharing committee on reasonable request after completion of a data-sharing request form and, if applicable, a data access agreement. Requests for data sharing in the first instance should be emailed to the corresponding author.
